# Burnout Syndrome in Occupational Therapists in Spain: Prevalence and Risk Factors

**DOI:** 10.3390/ijerph17093164

**Published:** 2020-05-02

**Authors:** Ana Cristina Escudero-Escudero, Antonio Segura-Fragoso, Pablo A. Cantero-Garlito

**Affiliations:** Faculty of Health Sciences, University of Castilla-La Mancha, 45600 Talavera de la Reina, Spain; escuderoescudero92@gmail.com (A.C.E.-E.); Antonio.Segura@uclm.es (A.S.-F.)

**Keywords:** burnout, occupational therapists, Maslach Burnout Inventory, healthcare professional

## Abstract

The objective of this study was to assess the potential levels of burnout syndrome in occupational therapists in Spain, as well as the risk factors involved in its prevalence. The data were obtained through an online survey. The tool utilised for this purpose was the Maslach Burnout Inventory General Survey (MBI-GS) that takes into consideration the following dimensions: emotional fatigue, depersonalisation and reduction of personal satisfaction. A sociodemographic questionnaire was also utilised. The sample of the study included 758 occupational therapists, 90.8% of whom were women and 9.2% of whom were men. For identifying the variables associated to the presence of burnout, the prevalence was compared through the chi-squared test, and the odds ratios by age were calculated through the binary logistic regression model. We found that 69.4% of the occupational therapists presented burnout syndrome and especially emotional fatigue (63.5%). Likewise, relevant and significant differences in the prevalence of burnout syndrome were observed depending on the age, marital status, number of children, work field and type of workday. We can conclude that burnout syndrome poses a clear risk for the health of occupational therapists that could have direct consequences on the work environment, affecting the way the interventions are performed with patients. This study could help to raise awareness on this reality and the factors implied. We suggest the implementation of measures (individual, labour and political) both for the prevention of burnout in occupational therapists as well as for reducing the levels of those who suffer from it.

## 1. Introduction

Burnout syndrome makes reference to “a syndrome of emotional fatigue, depersonalisation and reduction of personal satisfaction that can arise in people who work with people with any kind of disabilities” [1]. This is a response to events in the labour context over a prolonged time [2,3] that deploys itself through the manifestation of global signs and symptoms that vary depending on the person [3], emerging in an accumulative way and increasing progressively the severity of the symptoms [4]. It is characterised by three dimensions: emotional fatigue, depersonalisation of the treatment of people and difficulty for feeling a sense of reduction of personal satisfaction [2,5]. 

Burnout syndrome provokes serious consequences in the medium and long term at different levels [2]. At the individual level, it can cause chronic fatigue, cephalea, muscle soreness, sleeping disorders, hypertension, a higher incidence of alcohol and drugs abuse and a tendency to have accidents [6]. At a cognitive-emotional level, it creates a feeling of helplessness and failure, anxiety, difficulty concentrating and making decisions, self-esteem decrease, negative assessment and suicidal thoughts [2,6]. With regards to the socio-familiar level, deterioration of interpersonal relationships and an increase in family problems can be seen as well [2,7]. At an organisational level, some consequences are absenteeism, increase in staff rotations and deterioration of the quality of the services as well as the interventions performed by the occupational therapists themselves [5]. Likewise, burnout syndrome poses an increasing social and public health problem with an impact on the health of professionals [2,8,9] and implication of high social and economic costs [10].

The literature exposes numerous exhaustion sources for occupational therapy professionals. These sources come from the direct labour environment that conditions the professional, but they do not lie in the person itself [11]. These factors are grouped in two blocks related to the direct contact with the patients and to the environment [2]. These show characteristics such as fear of making any mistake, lack of appreciation and feedback on the part of the supervisors, overload of work, lack of professional identity and low visibility, patient behaviour, professional performance in chronic-care environments, lack of resources and lack of time among others [2,11,12,13,14]. Additionally, other aspects not related to work can simultaneously condition the professional for burnout syndrome. In this sense, greater levels of burnout have been shown in professionals with children in comparison with those who do not [15,16].

The work conditions that occupational therapists face every day are varied and occasionally inadequate: work overload, an excessive number of administrative tasks and lack of resources, among others [2]. These circumstances may cause burnout in a high percentage of occupational therapists [17], as reflected by the different works performed by this professional group. Research performed with Canadian therapists delivered strong results in the different levels of burnout, especially in cynicism (43.5%) and emotional fatigue (34.8%) [18]. A study with a sample of 126 participants in the State of Michigan revealed high values in the three dimensions of burnout related to the lack of professional identity [11]. Moreover, there are discrepancies depending on the work field. Although studies show differences, some refer to higher burnout in the occupational therapists who work in the psychosocial area than in those in the areas of physical and paediatric rehabilitation [19]. However, a recent study performed with 374 occupational therapists in Portugal has shown that therapists who work with children and older adults experience higher levels of burnout [4].

The goal of this research was to assess the potential levels of burnout among occupational therapists in Spain, and the risk factors involved in its prevalence.

## 2. Method

### 2.1. Participant Selection

The target population of this research was Spanish occupational therapists. The data were obtained through an online questionnaire. A strategy for choosing a sampling from different parts of the country was developed with the purpose of reaching the maximum heterogeneity possible. To this end, the maximum number of occupational therapists possible were contacted through emails that specified the aim of this study and the online questionnaire sent to all the professional colleges and associations of Occupational Therapy in Spain, encouraging all the collegiate members to take part in the research. The inclusion criterion in the study was that professionals had to be working in the same facility for at least six months.

### 2.2. Procedures

The Research Ethics Committee of the Integrated Health Area of Talavera de la Reina approved the study (Code CEIm 41/2018), as well as the documents of informed consent that the participants were provided. At all times, both the anonymity and the confidentiality of the answers of the participants were preserved. They were asked to take part in the study voluntarily. The questionnaires were administered between 15 January and 15 February 2019.

### 2.3. Tool

The tool utilised was the Maslach Burnout Inventory General Survey (MBI-GS) [20]—an inventory validated for the Spanish population for assessing the frequency and intensity of burnout syndrome. It consists of 22 items divided in three dimensions: cynicism and depersonalisation (5 items and a maximum score of 30); exhaustion or emotional fatigue (9 items and a maximum score of 54); and the reduction of personal satisfaction (8 items and a maximum score of 48). Following what has been established in different studies and research [21], the reference values established for determining signs of burnout lie in a score of more than 26 at emotional fatigue, more than 9 at depersonalisation and less than 33 at reduction of personal satisfaction. High scores in emotional fatigue and cynicism and low scores in professional efficiency correspond to the emergence of burnout syndrome [22]. Finally, different socio-demographic variables were collected: sex, age, marital status, number of children, work field, time in the work facility, type of contract and type of working hours.

### 2.4. Data Analysis

A descriptive analysis of the participant characteristics was carried out through absolute frequencies and percentages for each of the categories. The prevalence of burnout was described by percentages, with confidence intervals at 95% (CI 95%).

To identify the variables associated with the presence of burnout, the prevalence was compared through the chi-squared test and the odds ratios by age were calculated through the binary logistic regression model taking burnout as the dependent variable and the socio-demographic and work characteristics as independent variables with confidence intervals at 95%. As for contrasting the hypothesis, a level of significance of 5% (*p* < 0.05) was assumed. The statistical analysis was performed with the statistical software suite IBM-SPSS version 23.0 (SPSS Inc., Chicago, IL, USA). 

## 3. Results

The study sample included 758 occupational therapists, 688 of whom were women (90.8%) and 70 were men (9.2%). The majority of the sample (46.3%) were between 30 and 39 years of age. With reference to marital status, it was found that more than half of the sample, 435, were single (57.4%), and 224 (29.6%) were married. Additionally, a high proportion of participants, 514 (76.8%), had no children. 

The majority of the occupational therapists participating in the study, 327 (43.1%), were currently working with older adults, followed by 122 (16.1%) professionals who were working in the field of mental health. 

The most common interval of time working in the same facility among participants was between 1 and 2 years, with 225 (29.7%). 

The results show a great difference between the participants with permanent contracts and those with temporary or interim contracts; 525 (69.3%) occupational therapists were working permanently. Finally, with reference to the type of working hours, the sample shows that the number of professionals with morning shifts, 348 (45.9%), was essentially equal to the number of those with a split shift, 340 (44.9%) (see Table 1).

### Prevalence and Levels of Burnout

Table 2 shows the levels of burnout. The highest level corresponded to emotional fatigue, with 63.5%, followed by depersonalisation (33.9%). The lowest point obtained, with only 2.1%, corresponded to the reduction of personal satisfaction. It is important to note that 69.4% of the whole sample obtained positive results in burnout. Another relevant note is the great percentage of subjects who experienced emotional fatigue along with depersonalisation (26.1%).

Relevant and significant differences were observed in the prevalence of burnout depending on the age, marital status, number of children, work field and type of working hours (Table 3). A higher prevalence of burnout was associated with being younger, decreasing as the age increased. The level was higher than 70% in those under the age of 40, as opposed to the 57.9% in those above the age of 40. The ones who were single or had common law partners showed a higher prevalence (70–75%) than those who were married, divorced or widowed (50–58%). Those who did not have children showed higher prevalence (73.7%), and this decreased significantly and progressively when having just one child (63.7%) or two or more (57.7%). With regards to the work field, the lowest prevalence was observed among those who worked in the fields of physical rehabilitation (59.1%) or intellectual disability (63%), and the highest among those working with older adults (72.2%) and in childcare (71%). Another variable that obtained significant results was the type of working hours. Those with a split shift obtained the highest levels of burnout—75.3% of the people that had this type of shift showed higher burnout. 

No significant differences were observed in the prevalence of burnout depending on the gender, time working in the facility or type of contract—whether permanent, temporary or interim. 

## 4. Discussion

The results of this study showed that occupational therapists in Spain present high levels of burnout (69.4%), especially in the dimension of emotional fatigue and, to a lesser extent, in depersonalisation. These proportions are much higher than those found in the professionals of neighbouring countries such as Portugal (44%) [4] or the United Kingdom (32.54 %) [16].

Concerning age, the results obtained contrasted with those in other studies that suggested the older the professional, the higher their levels of burnout [4]. Another remarkable finding was that there were significant differences observed in the relationship between the levels of burnout and the marital status. Being single denoted higher levels of burnout than those who were married. Despite this, having a spouse and a family have previously been identified as potentially stressful factors [16]. On the other hand, in research carried out in 2002, no statistically significant differences were found in the results [15].

With regards to the number of children a participant had, the levels of burnout decreased as the number of children increased. However, these results were different to those obtained in other studies, where it was found that having children provoked higher levels of burnout than in those who had none [15,16].

Regarding the field of work, some studies have delivered the same results, verifying that there were no significant differences between the practical areas [4]; meanwhile, other research has delivered significant results in the fields of childhood [4] or physical rehabilitation and mental health [23], where higher scores were registered. 

Another relevant factor is the type of working hours, where it was found that the professionals with split shifts manifested a higher percentage of burnout than those who worked in morning or afternoon shifts. This finding was expected, and can be connected with another study where there was a significant correlation between the risk of exhaustion and prolonged work hours [24].

Likewise, we found no significant relationships with regards to gender, age, time working in the facility, work area or type of contract. With regards to the lack of significant findings in the gender and age factors, this is similar to former studies where no significant correlations were found in any of these demographic aspects [24,25].

Given these high results of burnout, it is necessary to take actions to address this situation. These strategies must be focused on the individual, the work context or the interaction between these two [4]: balancing workloads and ensuring equilibrium [5]; creating a healthy and safe environment by identifying the needs and the approach to problems with professional practises [26]; decreasing the number of treated cases daily [6]; dealing with stress, spending more time with family, maintaining an equilibrium between the personal and professional spheres and keeping the limits between both; improving the control of responsibilities; and keeping a good sense of humour and self-awareness, among others [18]. Likewise, it seems relevant to consider measures for improving the precariousness of the youngest occupational therapists. 

### 4.1. Limitations

This study provides a general perspective of burnout syndrome among the occupational therapists of Spain. However, there is no reliable data about the real number of occupational therapists performing this professional activity in different fields, so it is highly complex to establish the level of representativeness based on the number of therapists that have taken part in the survey with regards to the work field as a whole. On the other hand, work precariousness among therapists—especially among the youngest—makes them perform work in different fields and resources, making it difficult to gauge.

### 4.2. Implications for Occupational Therapy Practise

The findings of this study have the following implications for occupational therapy practise:

- Occupational therapists in Spain present very high levels of burnout. This work can help to create awareness on this reality and the factors that cause it in the face of its approach and the risk to health that it implies.
- There is a clear need for the implementation of measures (individual, work and political) both for the prevention of burnout in occupational therapists, as well as for reducing the levels of those who suffer from it already.

## 5. Conclusions

Burnout syndrome implies a clear risk for the health of occupational therapists, and it can have direct consequences on the work environment, affecting the way the interventions are performed with patients. This study can help to create awareness about this reality and the factors involved.

It would be interesting to delve into the levels of burnout depending on the fields where occupational therapists work in subsequent studies, as well as on the protective factors and the strategies utilised for facing the situations that cause burnout.

## Figures and Tables

**Table 1:** Characteristics of the people studied (*n* = 758).

		N°	%
Gender	Male	70	9.2%
	Female	688	90.8%
Age	<30 years old	274	36.1%
	30–39	351	46.3%
	40 years old or more	133	17.5%
Marital status	Married	224	29.6%
	Divorced, separated	22	2.9%
	Common law partner	75	9.9%
	Single	435	57.4%
	Widow/widower	2	0.2%
Number of children	None	514	67.8%
	1 child	102	13.5%
	2 or more children	142	18.7%
Work field	Elderly people	327	43.1%
	Mental health	122	16.1%
	Physical rehabilitation	115	15.2%
	Childcare	100	13.2%
	Intellectual disability	54	7.1%
	Other resources	40	5.3%
Time in the facility	Less than 1 year	87	11.5%
	1–2 years	225	29.7%
	3–5 years	128	16.9%
	6–8 years	98	12.9%
	9–11 years	117	15.4%
	12 years or more	103	13.6%
Type of contract	Permanent	525	69.3%
	Temporary	125	16.5%
	Interim	108	14.2%
Type of working hours	Morning shift	348	45.9%
	Split shift	340	44.9%
	Afternoon shift	70	9.2%

N°: Number of participants.

**Table 2:** Prevalence of burnout.

		N°	%	CI 95%
Emotional fatigue	Normal <= 26	277	36.5%	(33.10–40.03)
	Burnout > 26	481	63.5%	(59.91–66.80)
Depersonalisation	Normal <= 9	501	66.1%	(62.60–69.37)
	Burnout > 9	257	33.9%	(30.53–37.35)
Reduction personal satisfaction	Normal > 33	742	97.9%	(96.59–98.68)
	Burnout <= 33	16	2.1%	(1.211–3.400)
Burnout	NO	232	30.6%	(27.34–33.98)
	YES	526	69.4%	(65.97–72.56)
Burnout combinations	NO burnout	232	30.6%	(27.34–33.98)
	Just EF	267	35.2%	(31.82–38.69)
	Just D	45	5.9%	(4.362–7.853)
	Just RPS	0	0.0%	
	EF + D	198	26.1%	(23.02–29.36)
	EF + RPS	2	0.3%	(0.031–0.948)
	D + RPS	0	0.0%	
	EF + D + RPS	14	1.8%	(1.013–3.075)

CI 95%: confidence interval at 95%; EF: emotional fatigue; D: depersonalisation; RPS: reduction of personal satisfaction.

**Table 3:** Variables related to burnout.

			YES Burnout				Adjusted Per Age		
		N total	N	%	CI 95%	*p*	OR	CI 95%	*p* Adj Per Age
Gender	Male	70	49	70.0%	(57.86–79.45)	0.91	Ref		
	Female	688	477	69.3%	(65.73–72.66)		0.973	(0.567–1.670)	0.922
	Total	758	526	69.4%	(65.97–72.56)				
Age	<30 years old	274	201	73.4%	(67.70–78.24)	0.005	Ref		
	30–39	351	248	70.7%	(65.58–75.17)		1.813	(0.743–4.424)	0.191
	40 years old or more	133	77	57.9%	(49.03–65.95)		1.651	(0.888–3.068)	0.113
Marital status	Married	224	131	58.5%	(51.72–64.74)	<0.001	Ref		
	Divorced, separated	22	11	50.0%	(28.22–69.41)		1.379	(0.084–22.57)	0.822
	Common-law partner	75	53	70.7%	(59.02–79.75)		0.986	(0.054–17.91)	0.992
	Single	435	330	75.9%	(71.55–79.64)		2.343	(0.137–39.89)	0.556
	Widow/widower	2	1	50.0%	(1.257–90.57)		3.025	(0.180–50.57)	0.441
Number of children	None	514	379	73.7%	(69.70–77.35)	0.001	Ref		
	1 child	102	65	63.7%	(53.61–72.40)		1.889	(1.166–3.060)	0.010
	2 or more children	142	82	57.7%	(49.17–65.56)		1.241	(0.725–2.123)	0.431
Work field	Elderly people	327	236	72.2%	(66.97–76.74)	0.045	Ref		
	Mental health	122	84	68.9%	(59.83–76.38)		0.991	(0.551–1.780)	0.975
	Physical rehabilitation	115	68	59.1%	(49.57–67.68)		0.709	(0.360–1.396)	0.320
	Childcare	100	71	71.0%	(61.07–78.98)		2.134	(0.865–5.265)	0.100
	Intellectual disability	54	34	63.0%	(48.74–74.59)		1.037	(0.651–1.650)	0.877
	Other resources	40	33	82.5%	(67.22–91.17)		0.638	(0.372–1.091)	0.101
Time working in the facility	<1 year	87	57	65.5%	(54.55–74.66)	0.403	Ref		
	1–2 years	225	165	73.3%	(67.04–78.68)		0.604	(0.297–1.224)	0.162
	3–5 years	128	85	66.4%	(57.52–74.00)		0.942	(0.519–1.706)	0.843
	6–8 years	98	73	74.5%	(64.68–82.07)		0.746	(0.407–1.364)	0.341
	9–11 years	117	79	67.5%	(58.24–75.33)		1.198	(0.631–2.272)	0.581
	12 years or more	103	67	65.0%	(55.02–73.56)		0.893	(0.496–1.603)	0.704
Type of contract	Permanent	525	370	70.5%	(66.37–74.21)	0.622	Ref		
	Temporary	125	84	67.2%	(58.23–74.81)		0.879	(0.501–1.538)	0.651
	Interim	108	72	66.7%	(56.94–74.85)		1.143	(0.732–1.783)	0.557
Type of working hours	Morning shift	348	225	64.7%	(59.38–69.49)	0.006	Ref		
	Split shift	340	256	75.3%	(70.35–79.57)		1.152	(0.665–1.997)	0.613
	Afternoon shift	70	45	64.3%	(51.93–74.50)		1.731	(0.999–2.997)	0.050

N: Number of participants; *p*: *p*-value; OR: odds ratio; *p* adj per age: age-adjusted *p*-value.
